# Anticipated Regret, Entrepreneurial Cognition, and Entrepreneurial Persistence

**DOI:** 10.3389/fpsyg.2022.788694

**Published:** 2022-03-31

**Authors:** Mei-jiao Huang, Zhong-bin Li, Xiao-feng Su

**Affiliations:** ^1^College of Economics and Management, Fujian Agriculture and Forestry University, Fuzhou, China; ^2^College of Business Administration, Fujian Business University, Fuzhou, China

**Keywords:** anticipated regret, entrepreneurial cognition, entrepreneurial persistence, entrepreneurial environments, start-up enterprises

## Abstract

Based on the regret regulation theory and entrepreneurial cognition theory, this study aims to investigate the relationship between entrepreneurs’ anticipated regret and entrepreneurial persistence through the mediator of entrepreneurial cognition. To that end, we distributed surveys to entrepreneurs who were supported by the “Xing Chuang Tian Di” project and used 248 questionnaire data to examine the hypotheses. The results show that entrepreneurs’ anticipated regret has a significant and direct impact on entrepreneurial persistence. Also, entrepreneurship cognition plays a mediating role between anticipated regret and entrepreneurial persistence. In addition, the entrepreneurial environment plays a positive role in moderating the relationship between anticipated regret and entrepreneurial persistence.

## Introduction

Start-up enterprises have become a driving force of economic development in the world. However, start-up enterprises are usually confronted with such problems as weak anti-risk ability and low survival rate. The survival and development of start-up enterprises are related to whether entrepreneurs can adhere to the process of entrepreneurship, even if they encounter many difficulties (Cardon and Kirk, 2015). Previous researchers have taken entrepreneurial intention and entrepreneurial performance as outcome variables (Rauch et al., 2009; Hui et al., 2018) and analyzed the influence of entrepreneurial motivation, entrepreneurial characteristics, entrepreneurial competence, and social network on entrepreneurial intention and entrepreneurial performance (Taormina and Lao, 2007; Tatarko, 2013; Espiritu-Olmos and Sastre-Castillo, 2015; Nieuwoudt et al., 2017). Other studies have shown that positive psychological capital, social capital, human capital, and external environment may have an impact on the growth of start-up enterprises (Batjargal, 2003; Unger et al., 2011; Baluku et al., 2016; Sun et al., 2016). However, few people analyze entrepreneurial persistence and its influencing factors (Holland and Shepherd, 2013; Adomako et al., 2016). Entrepreneurship is a process of persistence, this process faces many risks and tasks, and entrepreneurs need to pay time and efforts to achieve the success of entrepreneurship (Zhu et al., 2018). Entrepreneurship persistence plays an important role in transforming entrepreneurial intentions into entrepreneurial behaviors and achieving entrepreneurial success ultimately (Mooradian et al., 2016). Entrepreneurial persistence is the behavior that entrepreneurs ignore adverse conditions, adversity, or other attractive choices, take entrepreneurial success as the goal orientation, and pursue entrepreneurial opportunities continuously (Holland, 2008).

Previous studies have shown that entrepreneurship is an emotional journey (Shepherd et al., 2015). Hayton and Cholakova (2012) explained the interplay relationship between emotions and cognitions in the stages of the entrepreneurial processes, and emotions would influence individual behaviors strongly in an uncertain environment (Cardon et al., 2012). Positive emotions such as enthusiasm, joy, and passion would promote entrepreneurial activities (Cardon et al., 2009; Welpe et al., 2012; Biraglia and Kadile, 2017; Shepherd and Patzelt, 2018). On the contrary, negative emotions such as fear and sadness would hinder entrepreneurial activities (Shepherd, 2003; Ning et al., 2017). However, emotions, no matter positive or negative, would not always play a beneficial or harmful role in the entrepreneurial process (Baron, 2008). In fact, some negative emotions such as fear, doubt, and aversion may also have a positive impact on the transformation of entrepreneurial intention into entrepreneurial behavior and encourage entrepreneurs to adapt themselves to entrepreneurial processes (Doern and Goss, 2014; Gelderen et al., 2015). Cognitive psychology theory holds that compared with positive emotions, negative emotions are related to different cognitive processes and behaviors (Forgas, 1995). Positive emotions would reduce individuals’ cognitive ability to think about entrepreneurship in a complex way and make it easy for them to ignore the key details in entrepreneurship (Dalborg and Wincent, 2015), while negative emotions make individuals pay more attention to the details in the uncertain and complex entrepreneurial environment.

Psychological studies reveal that emotional factors and counterfactual thinking such as anticipated regret can explain individuals’ intention and behavior (Gaglio and Katz, 2001). Hatak and Snellman (2017) concluded that anticipated regret exerts an important influence on behavioral regulation in early-stage entrepreneurship and fosters the transition from latent to nascent entrepreneurship. Anticipated regret is a negative emotional reaction that individuals experience as a result of comparing the anticipated outcome of their decision of not to act with the outcome they would have experienced if they had acted (Xiling et al., 2018). Anticipated regret moderates the intention-behavior relationship (Sheeran and Orbell, 1999). Anticipated regret has been explored in diverse disciplines such as marketing (Zeelenberg and Pieters, 2007), medicine (McEachan et al., 2016), real estate (Chen et al., 2011), and nascent entrepreneurship (Hatak and Snellman, 2017; Bouderbala, 2020). And still, no research has been made so far on the link of anticipated regret related to the entrepreneurship persistence behavior.

According to the theory of regret regulation, when anticipated regrets are generated, individuals will find ways to alleviate such regrets and will treat difficulties more carefully and think carefully. The clearer they know about the entrepreneurial environment and their entrepreneurial resource reserves, the better they obtain the resources needed in the entrepreneurial process consciously (Hatak and Snellman, 2017). Anticipated regret will affect entrepreneurs’ cognition and entrepreneurial behavior (Abraham and Sheeran, 2003), but how anticipated regret affects entrepreneurial persistence and what roles entrepreneurial cognition and entrepreneurial environment play in this process are not solved in the existing studies. Therefore, with the focus on the entrepreneurial stage (i.e., entrepreneurial action has been carried out rather than the formation stage of entrepreneurial intention) (Forbes and Milliken, 1999), the aim of this study was to demonstrate the effect of entrepreneurs’ anticipated regret on entrepreneurial persistence through the mediating role of entrepreneurial cognition and analyze the moderating role of entrepreneurial environment.

## Theoretical Basis

### The Theory of Regret Regulation

Anticipated regret means that individuals anticipate the disagreeable effects of what they might feel in case of regret and seek to protect themselves before they make a decision. Anticipated regret is defined as beliefs about feelings of regret or upset followed from inaction (Abraham and Sheeran, 2003), which refers to the negative emotional reaction that individuals experience when they imagine and compare the expected outcome of their decision of not to act with the outcome they would have experienced if they had acted (Loewenstein and Lerner, 2003). Anticipated regret is involved in behavioral regulation strategies and recognized as a motivational factor that expresses the feeling of motivating individuals to take action and hence to avoid experiencing regret arising from their failure to act. If entrepreneurs experience anticipated regret, they are encouraged to regulate this negative emotion *via* entrepreneurial activity to ameliorate the adverse effects of enduring regret on their psychological and physical health (Bouderbala, 2020).

The role of anticipated regret is drawn from the regret regulation theory (Pieters and Zeelenberg, 2007), which proposes that “regret is an cognitive emotion that people are motivated to regulate in order to maximize outcomes in the short-term and learn to maximize them in the long run” (Neneh, 2019). Accordingly, regret is subject to temporal reversal. In the short term, individuals regret their actions. But in the long term, they are likely to feel more intense regret over inaction if action is the norm (Pieters and Zeelenberg, 2007). Zeelenberg (1999) found that people always tend to make the choice of minimizing regret rather than minimizing risk. The result of this study solves why sometimes people tend to choose safety but sometimes they tend to take risks. The decision-maker is more willing to live in the *status quo* when making decisions. If the utility is reduced when choosing other schemes, an individual will regret more than reducing the same utility due to living in the *status quo*. As for the entrepreneurial process, entrepreneurs are more inclined to adhere to the choice of continuing the entrepreneurial process rather than giving up entrepreneurship, even if they may face the risk of entrepreneurial failure in the future.

### The Theory of Entrepreneurial Cognition

Since the 1980s, scholars have combined the theories of cognitive psychology and social cognition to focus on the impact of entrepreneurial cognition on entrepreneurial behaviors (Jun et al., 2015). The entrepreneurial cognitive theory explains the reasons for entrepreneurial success from the perspective of cognitive psychology and holds that entrepreneurs’ emotions will affect their cognitive process and then affect their entrepreneurial behavior (Gendolla, 2000; Mitchell et al., 2002, 2007; Baron, 2004; Bird et al., 2012). Entrepreneurs’ positive or negative emotions affect entrepreneurs’ cognitive structure and cognitive process (Byrne and Shepherd, 2015). At present, the studies on entrepreneurial cognition take “situation-thinking behavior” as the basic framework to explore “how the situation affects entrepreneurs’ cognition and decision-making process and then leads to different behavior results” (Mitchell et al., 2007). Entrepreneurial cognition reflects the rational thinking process and predicts entrepreneurial behavior effectively (Buckley and Halbesleben, 2017; Randolph-Seng et al., 2020).

The researchers of entrepreneurial cognition are different from psychology and behavioral science because the uniqueness of entrepreneurial cognition comes from entrepreneurial situations rather than entrepreneurs. The theory focuses on exploring the applicability and dynamics of existing knowledge and styles about cognitive processes in entrepreneurial situations (Baron, 2004). Mitchell et al. (2002) considered that entrepreneurial cognition is the knowledge structure for entrepreneurs to make evaluation, judgment, and decision in the process of opportunity evaluation and entrepreneurial enterprise growth, including three scripts, namely, preparation script, willingness script, and ability script. The script is a highly developed ordered knowledge (He et al., 2021), which can shape individual action-based knowledge structure. Preparation script refers to the necessary factors to form a new enterprise, such as relationships, resources, and property. Willingness script refers to the commitment to risk and the perception of entrepreneurship ideas. Ability script refers to the skills, knowledge, norms, and attitudes required for entrepreneurial success (Doern and Goss, 2013; Jun et al., 2015; Randolph-Seng et al., 2015). Entrepreneurs with rich entrepreneurial cognition can encode the perceived information in a more complex way. Entrepreneurs who have sufficient entrepreneurial preparations, greater willingness to start a business, and higher levels of entrepreneurial ability can identify entrepreneurial business opportunities by piecing useful information and resources together from the experience (Mitchell et al., 2002) and respond to the problems in the process of starting a business actively.

## Hypothesis Development and Research Model

### The Influence of Anticipated Regret on Entrepreneurial Persistence

In entrepreneurship, it is better to understand the impact of regret not as a consequence of not following plans to create a venture but as a counterfactual and anticipatory negative emotion that drives entrepreneurs to regulate their behavior and is more likely to move to the later phase of the entrepreneur’s process. The regret regulation theory postulates that individuals are regret averse and try to regulate their regrets to maximize outcomes in the long run. Anticipated regret plays an important role in transforming the original entrepreneurial actions into follow-up entrepreneurial persistence. Thus, anticipated regret manifests as a feeling for doing by motivating individuals to take action to avoid experiencing regret arising from a failure to act (Zeelenberg and Pieters, 2007). In fact, in contrast with other negative emotions, anticipated regret can help people gain insights into themselves (Kuhnle et al., 2014) and act as a behavioral push toward engaging in business activities.

Entrepreneurs with higher anticipated regret are more likely to conduct counterfactual behavior analysis and give full consideration to the consequences of giving up entrepreneurship (Hatak and Snellman, 2017). Reducing the regret over not following plans for an entrepreneurial venture drives entrepreneurs to regulate their behavior in such a way that they are more likely to proceed to persistence. By engaging in actual start-up behavior, the nascent entrepreneur regulates the regret that would arise from a failure to act, thus establishing grounds for maximizing the uncertain but socially important start-up outcomes in the long run (Hatak and Snellman, 2017). Conner et al. (2015) recognized a predictive role associated with the anticipated affect that can account for up to 18.9% of intention and 9.9% of behavior. In other words, individuals who think about entrepreneurial action positively (latent entrepreneurs), while at the same time perceiving entrepreneurial inaction as regrettable (Hird, 2007), will engage in business gestation activities to minimize future regrets, whereas those who do not associate entrepreneurial inactivity with anticipated regret are less likely to persistence. Based on the abovementioned analysis, we proposed the following hypothesis:

Hypothesis 1: Anticipated regret has a significant positive effect on entrepreneurial persistence.

### The Relationship Between Anticipated Regret and Entrepreneurial Cognition

Entrepreneurs’ emotions affect entrepreneurial actions by adjusting their cognitive abilities and cognitive processes (Liguori et al., 2018). Anticipated regret stimulates to compile information and to orient the cognitive process. Anticipated regret constitutes a non-rational belief in a future prospect. It simultaneously triggers regretful thinking, i.e., the automatically activated cognitive representation of alternative scenarios. The regret regulation theory holds that entrepreneurs will pay more attention to the consideration of entrepreneurial details and self-improvement to reduce the anticipated regret caused by giving up starting a business, thus optimizing the entrepreneurial cognitive structure. These entrepreneurs will make more efforts to integrate all kinds of knowledge, collect all kinds of information about their industries, markets, and competitors to understand entrepreneurial risks, and seek human support, financial support, and even emotional support related to entrepreneurship in a variety of ways. In turn, these will optimize the composition of entrepreneurial cognition from three aspects, namely, preparation script, willingness script, and ability script (Mitchell et al., 2002; Randolph-Seng et al., 2015), and then better identify new products or services to ensure the sustainability of entrepreneurial activities. Accordingly, we proposed the following hypotheses:

Hypothesis 2: Anticipated regret has a positive effect on entrepreneurial cognition.
Hypothesis 2a: Anticipated regret has a positive effect on preparation script.
Hypothesis 2b: Anticipated regret has a positive effect on willingness script.
Hypothesis 2c: Anticipated regret has a positive effect on ability script.

### The Relationship Between Entrepreneurial Cognition and Entrepreneurial Persistence

Based on the entrepreneurial cognitive theory, entrepreneurial cognition affect entrepreneurial behaviors (Heilman et al., 2010), and different entrepreneurial cognitive structures will lead to different entrepreneurial behaviors (Baron, 2004). Entrepreneurs who have sufficient entrepreneurial preparations, greater entrepreneurial willingness, and higher levels of entrepreneurial ability will be easier to identify entrepreneurial opportunities and deal with difficulties in the entrepreneurial process (Oyson and Whittaker, 2015). With better entrepreneurial cognition, entrepreneurs are more likely to make correct entrepreneurial decisions, gain more entrepreneurial support, and obtain higher entrepreneurial performance (Foo et al., 2014), which will lead entrepreneurs to persist in starting a business (Walsh and Elorriaga-Rubio, 2019). Gong and Yang (2021) hold that the richer the knowledge structure of various resources, willingness, and abilities that exist in the minds of entrepreneurs, the higher the cognitive level of entrepreneurs, so they are more likely to carry out persistence behavior automatically. We believe that in the context of entrepreneurial persistence, the three dimensions of entrepreneurial cognition are closely related and play their respective roles. The preparation script explains the resource conditions of persistence behavior, the willingness script explains the internal driving force of entrepreneurship persistence, and the ability script explains the ability conditions of entrepreneurs to persist in entrepreneurship. Therefore, a higher level of entrepreneurial willingness, more entrepreneurial preparation, and a higher level of entrepreneurial ability will help entrepreneurial persistence (Cardon and Kirk, 2015). Accordingly, we propose the following research hypotheses:

Hypothesis 3: Entrepreneurial cognition has a positive effect on entrepreneurial persistence.
Hypothesis 3a: Preparation script has a positive effect on entrepreneurial persistence.
Hypothesis 3b: Willingness script has a positive effect on entrepreneurial persistence.
Hypothesis 3c: Ability script has a positive effect on entrepreneurial persistence.

### The Mediating Role of Entrepreneurial Cognition

Entrepreneurial persistence will be affected by entrepreneurial cognition, and anticipated regret will affect entrepreneurial persistence through entrepreneurial cognition. Based on the theory of entrepreneurial cognition, entrepreneurial cognition plays a mediating role between entrepreneurial emotions and entrepreneurial behaviors (Liguori et al., 2018). The negative emotions such as anticipated regret will enhance the cognitive ability of individuals to think about entrepreneurship in a complex way, constantly promote entrepreneurs to break the original mental models, and make entrepreneurs better understand the positive and negative information in the process of entrepreneurship. As entrepreneurs face many complicated entrepreneurial tasks or attractive business opportunities in the middle and late stages of in-depth entrepreneurial activities, according to the regret adjustment theory, after choosing entrepreneurship, entrepreneurs tend to adjust the composition of entrepreneurial cognitive structure through information collection, resource acquisition, and ability improvement, so as to adhere to entrepreneurial activities. Anticipated regret makes the entrepreneur consider the different details of the start-up process carefully and adhere to the entrepreneurial behavior rationally. In order to reduce the anticipated regret caused by giving up starting a business, many entrepreneurs will adopt effective strategies to regulate their behaviors (Xuejun and Chunguo, 2020), such as collecting a large amount of positive entrepreneurial information, improving entrepreneurial skills, and changing the entrepreneurial cognitive structure (Adomako et al., 2016). Those with lower anticipated regret are unlikely to obtain more entrepreneurial information and resources to persist in entrepreneurship (Hatak and Snellman, 2017). Based on this, this study puts forward the fourth hypothesis as follows:

Hypothesis 4: Entrepreneurial cognition plays a mediating role between anticipated regret and entrepreneurial persistence behavior.

### The Moderating Role of Entrepreneurial Environment

Whether entrepreneurial activities can be carried out smoothly or not will be affected by a series of external environments such as infrastructure, natural ecology, policy support, and entrepreneurial atmosphere. The values of the entrepreneurial environment perceived by entrepreneurs are important to adhere to entrepreneurship (Peng et al., 2021). When the Internet, communication equipment, and transportation facilities are complete, the ecology is excellent, the entrepreneurial policy is supported strongly, and the entrepreneurial atmosphere is good, it will be easier for entrepreneurs to identify entrepreneurial opportunities and get more entrepreneurial materials and financial supports. Social cognitive theory holds that the entrepreneurial environment will affect entrepreneurs’ mood and behaviors (Luthans et al., 2000; Gomezelj and Kusce, 2013), and the perception of the entrepreneurial environment will directly stimulate their evaluation of the current situation and future of entrepreneurship. The richer and better the entrepreneurial environment is, the more satisfied with the external environment the entrepreneurs will be. The better the entrepreneurial environment is, the stronger the anticipated regrets are, and the more reluctant to give up continuing to start a business they will be. Therefore, even if the entrepreneurial process is complicated and difficult, in the context of a good entrepreneurial environment, they are more willing to choose to persist in entrepreneurship. The stronger their anticipated regrets are, the more willing they are to continue their entrepreneurial activities. Based on this, the following assumption is proposed:

Hypothesis 5: Entrepreneurial environment plays a moderating role between anticipated regret and entrepreneurial persistence.

**FIGURE 1:**
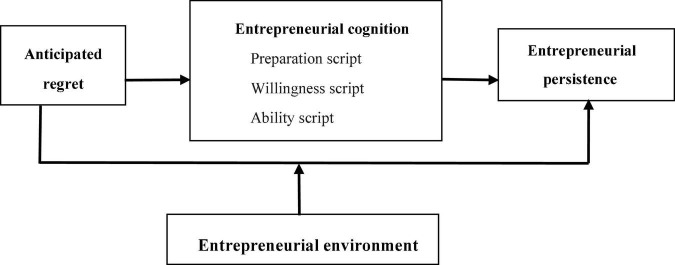
Research model.

Based on the abovementioned analysis, this study builds a theoretical model of anticipated regret, entrepreneurial cognition, entrepreneurial persistence, and entrepreneurial environment as shown in Figure 1.

## Materials and Methods

### Participants and Procedure

A field survey approach was employed in this study. Our survey was performed in the training class of the Fujian Entrepreneurship Center in 2019 and 2020. The entrepreneurs who participated in the training course were all financially supported by the “Xing Chuang Tian Di” project. During our investigation, we found that many of the entrepreneurs are farmers or entrepreneurs who worked in cities and then returned to their hometown to start a business. These entrepreneurs played an important role in the implementation of the strategy of Rural Revitalization and the tackling of poverty in China. This study analyzes their entrepreneurial persistence, which is of great significance to help rural economic development.

The questionnaires were distributed to entrepreneurs during the classes with instructors’ help. A detailed explanation of the research purposes was given to the participants. On completion, the questionnaires were immediately collected. First, a pilot test was conducted prior to the formal investigation, and 151 respondents completed the questionnaires, 146 were returned, and 101 valid questionnaires remained after questionnaire screening to verify the readability and clarity of all the measurement items. Outliers include those whose entrepreneurial enterprises were established for more than 8 years. This ensures the internal consistency of our sample. Second, a total of 325 were distributed for the formal investigation. Of the 325 cases, 248 responses were used for data analysis after the removal of outliers and invalid data. Finally, the data were screened for the characteristics of unengaged responses. Cases with an SD less than 0.5 were subject to an investigation for potential unengaged responses.

Participants’ demographic data were as follows: 97 (39.1%) were women and 151 (60.9%) were men. In terms of entrepreneurs’ age, 22 (8.9%) were aged 20 years and below, 106 (42.7%) were aged 21–30 years, 89 (35.9%) were aged 31–40 years, 29 (11.7%) were aged 41–50 years, and 2 (0.8%) were aged above 51 years. No entrepreneurs were educated in elementary school and below, 38 (15.3%) were educated in junior high school, 89 (35.9%) were educated in high school (or middle school), 25 (25%) were educated in university, and 59 (23.8%) were educated as graduate students. Regarding work years before entrepreneurship, 13 (5.2%) at least had 1 year of working experience, 37 (14.9%) had 2–5 years of working experience, 88 (35.5%) had 6–10 years of working experience, 74 (29.8%) had 11–15 years of working experience, and 36 (14.5%) had above 15 years of working experience. In terms of the years of these enterprises, 43 (17.3%) were established at least 1 year, 95 (38.3%) were established 1–2 years, 91 (36.7%) were established 3–4 years, 17 (6.9%) were established 5–6 years, and 2 (0.8%) were established 7–8 years.

### Measures

**FIGURE 2:**
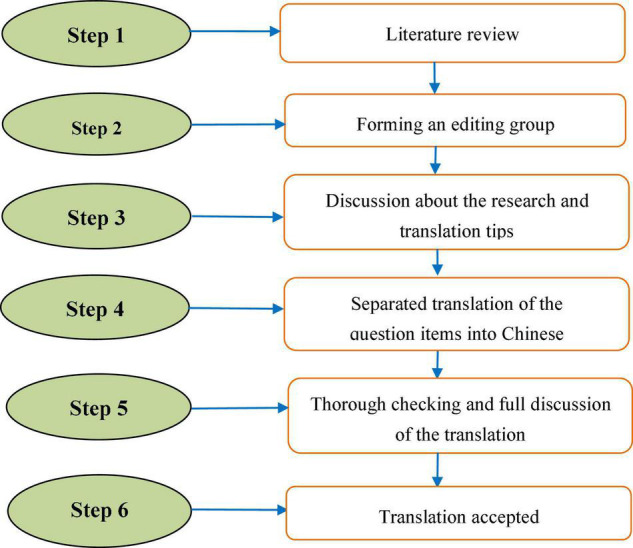
Questionnaire designing procedure.

In this study, the measures for study variables were adopted from the existing literature. Because some of the original questionnaire items were written in English, we need to make sure that the items should be translated appropriately and understandable for Chinese respondents. Before translating the questionnaire items to English version, we formed an editing group that consists of five members. Among whom, three members are university professors with the same research field of entrepreneurship. Then, two overseas students were invited to participate in the group. They are both native English speakers and also have a good command of Chinese. Hence, their participation was beneficial for the translation and revision of the questionnaire items. First of all, the authors had a discussion with the two overseas students about the aim of the research and the requirements for the translation of questionnaire items. Then, they were asked to translate the questionnaire items into a Chinese version separately. After that, their translations were thoroughly checked by the three professors in order to ensure the content validity of the items. They also offered suggestions to add, remove, or revise inappropriate items. Finally, the checked translation was further discussed by the whole editing group. The revised version could not be accepted until the whole editing group had reached an agreement on it. The questionnaire designing procedure is shown in Figure 2.

Also, a 5-point Likert-type scale ranging from 1 (extremely disagree) to 5 (extremely agree) was utilized to measure anticipated regret, entrepreneurial cognition, entrepreneurial persistence, and entrepreneurial environment.

With regard to anticipated regret, which is considered as an independent antecedent of intention, it is recommended to measure in relation to not performing a behavior, whereas attitudes, subjective norms, and perceptions of control are assessed in relation to performing it (Ajzen and Sheikh, 2013). Combined with the situation in China and the suggestions of three entrepreneurship experts, we added an item based on the measurement of anticipated regret in the previous literature research (Abraham and Sheeran, 2004; Ajzen and Sheikh, 2013). Hence, it was measured by three questionnaire items, namely, “If I don’t continue to engage in entrepreneurial activities in the next 12 months, I will regret it,” “If I don’t continue to engage in entrepreneurial activities in the next 12 months, I will be frustrated,” and “If I don’t continue my entrepreneurial activities in the next 12 months, I will feel anxious.” All measurement items have passed the readability and clarity in the pilot test.

Entrepreneurial cognition was measured with 18 items from the classification proposed by Mitchell et al. (2002). Of which, five items were for the preparation script. The example items are “I have the resources to start a business” and “I can easily enter the field ready to start a business.” Five items were for the willingness script, and the example items are “I can quickly adapt to the new environment” and “I’m always ready for change.” Five items were for the ability script, and the example items are “I can accurately identify potential business opportunities” and “I have a good entrepreneurial knowledge reserve.” All measurement items have passed the readability and clarity in the pilot test.

To measure entrepreneurial persistence, we used six items from the study by Baum et al. (2001), such as “I will continue to work hard to start a business even if others oppose me,” “No matter how challenging and difficult my business is, I will not give up,” and “Compared with most other people, I put more effort into entrepreneurship.” Five measurement items have passed the readability and clarity in the pilot test, but one item failed, so we deleted the item. Finally, five measurement items left.

The entrepreneurial environment was measured with 5 items from the classification proposed by Liu (2011), e.g., “I think the financial service environment is good” and “I think the social and economic environment in my hometown is good.” All measurement items have passed the readability and clarity.

Considering the influence of the control variable, we took gender, age, previous working years (W experience), educational background, and entrepreneurial experience (E experience) as control variables.

## Results

### Reliability and Validity

We conducted reliability and confirmatory factor analyses (Table 1). All the Cronbach’s alpha values were above 0.90, and the factor loadings, composite reliabilities (CRs), and the average variance extracted (AVE) values were higher than their recommended coefficient weights (Fornell and Larcker, 1981).

**TABLE 1:** Convergent validity of the measurement model.

Variable	Questionnaire item	Factor loading	CR	AVE	Cronbach’s alpha
Anticipated regret (AR)	AR1	0.885	0.915	0.783	0.923
	AR2	0.895			
	AR3	0.874			
Willingness script (WS)	WS1	0.844	0.934	0.738	0.962
	WS2	0.851			
	WS3	0.856			
	WS4	0.844			
	WS5	0.898			
Preparation script (PS)	PS6	0.884	0.950	0.791	0.949
	PS7	0.881			
	PS8	0.912			
	PS9	0.848			
	PS10	0.921			
Ability script (AS)	AS11	0.796	0.920	0.698	0.95
	AS12	0.863			
	AS13	0.829			
	AS14	0.809			
	AS15	0.878			
Entrepreneurial environment (EE)	EE1	0.854	0.944	0.772	0.935
	EE2	0.913			
	EE3	0.84			
	EE4	0.907			
	EE5	0.876			
Entrepreneurial persistence (EP)	EP1	0.808	0.934	0.739	0.961
	EP2	0.864			
	EP3	0.872			
	EP4	0.867			
	EP5	0.884			

### Correlation Analysis of Variables

As shown in Table 2, the bold numbers in the diagonal direction represent the square roots of AVEs. Because the square roots of AVEs in the diagonal direction are all greater than the off-diagonal numbers, the discriminant validity is satisfactory for all variables. In addition, there are significant correlations between anticipated regret, entrepreneurial cognition, entrepreneurial persistence, and entrepreneurial environment. The relationship between the four variables needs to be deeply explored.

**TABLE 2:** Pearson’s correlation coefficient matrix for study variables.

Variable	AVE	AR	EP	WS	PS	AS	EE
AR	0.783	**0.885**					
EP	0.738	0.463**	**0.859**				
WS	0.791	0.192*	0.419**	**0.889**			
PS	0.698	0.313**	0.328**	0.210**	**0.835**		
AS	0.772	0.170**	0.415**	0.655**	0.231**	**0.879**	
EE	0.739	0.147*	0.304**	0.149*	0.194**	0.139*	**0.859**

*AR, anticipated regret; EP, entrepreneurial persistence; WS, willingness script; PS, preparation script; AS, ability script; EE, entrepreneurial environment. The items on the diagonal on bold represent the square roots of the AVE; off-diagonal elements are the correlation estimates.*

** indicates significant level of two-tailed test p < 0.01, * indicates significant level of two-tailed test p < 0.05.

### Hypotheses Testing

In this study, SPSS software was used for regression analysis. The hierarchical regression method was used to verify relevant research hypotheses. First, the results of the regression test of anticipated regret and entrepreneurial persistence are shown in M1 in Table 3. It shows that anticipated regret has a significantly positive impact on entrepreneurial persistence (β = 0.434, *p* < 0.001), thus Hypothesis 1 is supported. Second, from M2, willingness script (β = 0.211, *p* < 0.01), preparation script (β = 0.231, *p* < 0.001), and ability script (β = 0.2, *p* < 0.01) significantly affect entrepreneurial persistence. From M3, it can be found that entrepreneurial cognition (β = 0.639, *p* < 0.001) has a significantly positive impact on entrepreneurial persistence, assuming that Hypothesis 2a, Hypothesis 2b, Hypothesis 2c, and Hypothesis 2 are supported. Then, from M4, M5, and M6 in Table 3, it can be noted that anticipated regret significantly affects willingness script (β = 0.187, *p* < 0.01), preparation script (β = 0.319, *p* < 0.001), and ability script (β = 0.15, *p* < 0.05), assuming that Hypothesis 3a, Hypothesis 3b, and Hypothesis 3c are supported. Also, the non-standardized regression coefficient of the anticipated regret on entrepreneurial cognition is 0.219 (*p* < 0.001), which shows that Hypothesis 3 is supported.

**TABLE 3:** Regression results of anticipated regret, entrepreneurial cognition, and entrepreneurial persistence.

Variable	Entrepreneurial persistence (EP)			Entrepreneurial cognition (EC)			
				WS	PS	AS	EC
	M1	M2	M3	M4	M5	M6	M7
Anticipated regret	0.434***			0.187**	0.319***	0.15*	0.219***
Willingness script (WS)		0.211**					
Preparation script (PS)		0.231***					
Ability script (AS)		0.200**					
Entrepreneurial cognition			0.639***				
Gender	0.051	0.026	0.026	0.040	0.018	–0.016	0.014
Age	–0.109	–0.090	–0.087	–0.178	0.057	–0.148	–0.090
Education	0.017	0.062	0.059	0.008	–0.130	0.037	–0.028
W experience	0.065	0.090	0.087	0.101	–0.097	0.114	0.039
E experience	–0.016	–0.005	–0.006	–0.007	–0.063	0.002	–0.023
*R* ^2^	0.222	0.268	0.268	0.053	0.114	0.041	0.100
Adj. *R*^2^	0.203	0.244	0.250	0.030	0.092	0.017	0.077
*F*	11.490	10.957	14.700	2.253	5.191	1.731	4.440

*p < 0.05; **p < 0.01; ***p < 0.001.

The analysis of mediating effect was conducted according to the processes proposed by Zhao et al. (2010). To verify the mediating effect of entrepreneurial cognition, it is necessary to regress the anticipated regret and entrepreneurial cognition to the entrepreneurial persistence at the same time. According to the results of the data in Table 4, compared with the corresponding results of the data in Table 3, the correlation coefficients of anticipated regrets and entrepreneurial cognition have decreased, refer to M8 (β = 0.376 < 0.434, *p* < 0.001), M9 (β = 0.37 < 0.434, *p* < 0.001), and M10 (β = 0.383 < 0.434, *p* < 0.001). It can be noted from M11 that the correlation coefficient of anticipated regret drops to 0.322 (*p* < 0.001), indicating that entrepreneurial cognition plays a mediating role between anticipated regret and entrepreneurial persistence. Hence, Hypothesis 4 is supported. Therefore, the more anticipated regret the entrepreneurs have, the more they tend to look for better entrepreneurial information, thus bringing more entrepreneurial activities.

**TABLE 4:** Results of the regression analysis of entrepreneurial cognitive mediation.

Variable	Entrepreneurial persistence			
	M8	M9	M10	M11
Anticipated regret	0.376***	0.370***	0.383***	0.322***
Willingness script	0.313***			
Preparation script		0.202***		
Ability script			0.341***	
Entrepreneurial cognition				0.514***
Gender	0.038	0.047	0.056	0.044
Age	–0.053	–0.120	–0.058	–0.063
Education	0.014	0.043	0.004	0.031
W experience	0.034	0.085	0.026	0.045
E experience	–0.014	–0.004	–0.017	–0.005
*R* ^2^	0.330	0.261	0.334	0.370
Adj. *R*^2^	0.310	0.239	0.314	0.352
*F*	16.864	12.112	17.188	20.138

***p < 0.001.

**FIGURE 3:**
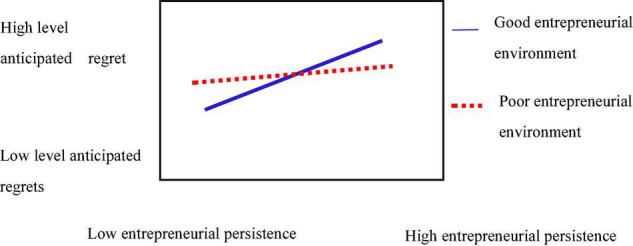
Moderating effect of entrepreneurial environment between anticipated regret and entrepreneurial persistence.

The hierarchical regression method was used to examine the moderating effect of entrepreneurial environment between anticipated regret and entrepreneurial persistence. The regression results are shown in Table 5. The moderation effect of anticipated regret×entrepreneurial environment on entrepreneurial persistence is 0.15 (*t* = | 3.276| > 1.96, *p* < 0.01), indicating the existence of a moderation effect, representing that every unit increases in the moderation variable of entrepreneurial environment, the slope of anticipated regret on entrepreneurial persistence increases by 0.15 units, and Hypothesis 5 is supported. The simple slope analysis in Figure 3 shows that the entrepreneurial environment plays a significant moderating effect between anticipated regret and entrepreneurial persistence. Therefore, the better the entrepreneurial environment, the more anticipated regret the entrepreneurs have, and the more they tend to persist to achieve entrepreneurial success.

**TABLE 5:** Results of the moderating effect test.

Variable	Entrepreneurial persistence		
	M1	M11	M12
Anticipated regret	0.434***	0.400***	0.363***
Entrepreneurial environment		0.238***	0.218***
Anticipatory regret×Entrepreneurial environment			0.150**
Gender	0.051	0.087	0.090
Age	–0.109	–0.093	–0.097
Education	0.017	0.023	0.013
W experience	0.065	0.050	0.047
E experience	–0.016	–0.043	–0.041
*R* ^2^	0.222	0.282	0.313
Adj. *R*^2^	0.203	0.261	0.290
*F*	11.49	13.459	13.596

**p < 0.01; ***p < 0.001.

## Discussion

Previous researchers have found that the positive effects of positive emotions and the negative effects of negative emotions in the process of starting a business (Grichnik et al., 2010; Patzelt and Shepherd, 2011; Su et al., 2020) ignored the positive effects of many negative emotions in the entrepreneurial process. Based on the regret adjustment theory, we added anticipated regret into the research field of entrepreneurship and analyzed its positive impact on entrepreneurial persistence, because anticipated regret encourages entrepreneurs to regulate their behavior after entering the entrepreneurial process to reduce their regrets. Hatak and Snellman (2017) pointed out that anticipated regret has a positive impact on entrepreneurial behavior but did not point out how anticipated regret affects entrepreneurial persistence. Our study incorporated anticipated regret into entrepreneurship research and found the positive effects of entrepreneurs’ anticipated regret on entrepreneurial persistence through the mediating role of entrepreneurial cognition, and the entrepreneurial environment plays a positive moderating effect.

This study focuses on the process of the actual entrepreneurial behavior, rather than the process from entrepreneurial intention to entrepreneurial action. The reason why most people still insist on entrepreneurial behavior even when they encounter difficulties is that anticipated regret plays a positive stimulating role. This study makes several contributions to the literature. First, as entrepreneurial persistence is the key to the success of entrepreneurship (Adomako et al., 2016), our study analyzed entrepreneurial persistence as an outcome variable, which is a meaningful attempt. Second, our study has identified the positive effects of negative emotions and found that anticipated regret can help entrepreneurs better understand the current status and future of entrepreneurship. The stronger the feeling of “regrets if don’t do it,” the more it will promote entrepreneurial persistence. This study enriches our understanding of whether and how negative emotions such as anticipated regret lead to subsequent behavior. Third, our study found that entrepreneurial cognition has a positive effect on entrepreneurial persistence, the preparation script provides the resource conditions, the willingness script provides the motive force, and the ability script provides the ability conditions, which are the important foundation of entrepreneurs to persist in entrepreneurship. Fourth, our study found that entrepreneurial cognition plays a mediating role between anticipated regret and entrepreneurial persistence, which shows that anticipated regret can affect entrepreneurial persistence by affecting entrepreneurial cognition. Fifth, the results of the data show that the entrepreneurial environment plays a positive moderating role between anticipated regret and entrepreneurial persistence, i.e., the better the entrepreneurial environment, the more beneficial it is for entrepreneurs to reduce their anticipated regrets by persisting in entrepreneurship.

Based on these research conclusions, the following practical suggestions are given to entrepreneurs and entrepreneurial policymakers. Entrepreneurs will experience a variety of positive or negative emotions in the process of entrepreneurship. On the one hand, entrepreneurs should understand the impact of emotions objectively (Karimi, 2020), learn to manage emotions, and pay attention to the positive effects of negative emotions. On the other hand, entrepreneurs should improve their entrepreneurial cognition level so as to better understand the entrepreneurial process and think rationally, and they do not ignore the blind insistence on entrepreneurship that anticipated regret may bring (Holland and Shepherd, 2013). Furthermore, entrepreneurs must fully understand and use the favorable conditions in the entrepreneurial environment to obtain more social support. Relevant entrepreneurship support policies can appropriately join emotional work and set up certain guidance to help entrepreneurs understand the meaning of anticipated regret correctly (Bjälkebring et al., 2014). When entrepreneurs experience entrepreneurial failure and feel “regrets after doing it,” relevant entrepreneurial mentors can provide some strategies to deal with the negative emotions. In addition, entrepreneurship support policies can be included, such as effective entrepreneurship training courses, lectures, and forums. Valuable guidance from experts and knowledge sharing from entrepreneurs can not only improve entrepreneurs’ entrepreneurial cognition level (Barazandeh et al., 2015; Westhead and Solesvik, 2015) but also promote their psychological safety (He et al., 2020). In addition, the local government can create a good entrepreneurial atmosphere and can provide facilities and institutional support.

Despite many contributions to the literature, this study also has some limitations. First, the center of this study is a direct driver of anticipated regret for entrepreneurial persistence. However, there are many other positive emotional factors (e.g., entrepreneurial passion and entrepreneurial self-efficacy) that are not taken into account in our theoretical framework. In future research, it is recommended to broaden the proposed theoretical model by incorporating them. The second limitation is that this study has lies in the geographic focus. Specifically, the samples of this study were some entrepreneurs supported by the “Xing Chuang Tian Di” project in Fujian Province. Further studies should be conducted to examine the general entrepreneurs and use different samples from other geographical areas. Third, we only consider the characteristics of entrepreneurs as the control variables, there are many factors that affect entrepreneurial persistence such as enterprise-scale, industry, and performance. Thus, we recommend that future researchers may consider the impact on relevant variables from the perspectives of individuals, organizations, and the external environment as far as possible.

## Data Availability Statement

The raw data supporting the conclusions of this article will be made available by the authors, without undue reservation.

## Ethics Statement

Ethical review and approval were not required for the study on human participants in accordance with the Local Legislation. Written informed consent from the participants was not required to participate in this study in accordance with the national legislation and the institutional requirements.

## Author Contributions

M-JH and Z-BL: conceptualization. M-JH: methodology investigation, data curation, analysis, writing – original draft preparation. M-JH, Z-BL, and X-FS: validation, formal analysis, and writing – review and editing. M-JH: supervision and funding acquisition. All authors have read and agreed to the published version of the manuscript.

## Conflict of Interest

The authors declare that the research was conducted in the absence of any commercial or financial relationships that could be construed as a potential conflict of interest.

## Publisher’s Note

All claims expressed in this article are solely those of the authors and do not necessarily represent those of their affiliated organizations, or those of the publisher, the editors and the reviewers. Any product that may be evaluated in this article, or claim that may be made by its manufacturer, is not guaranteed or endorsed by the publisher.
